# Skeletal Muscle Depletion Predicts the Prognosis of Patients with Hepatocellular Carcinoma Treated with Sorafenib

**DOI:** 10.3390/ijms16059612

**Published:** 2015-04-28

**Authors:** Kenji Imai, Koji Takai, Tatsunori Hanai, Takayasu Ideta, Tsuneyuki Miyazaki, Takahiro Kochi, Atsushi Suetsugu, Makoto Shiraki, Masahito Shimizu

**Affiliations:** Department of Gastroenterology/Internal Medicine, Gifu University Graduate School of Medicine, 1-1 Yanagido, Gifu 501-1194, Japan; E-Mails: koz@gifu-u.ac.jp (K.T.); hanai0606@yahoo.co.jp (T.H.); taka.mailbox.789@gmail.com (T.I.); tsunemiyazaking@yahoo.co.jp (T.M.); kottii924@gmail.com (T.K.); asue327@yahoo.co.jp (A.S.); mshiraki-gif@umin.ac.jp (M.S.); shimim-gif@umin.ac.jp (M.S.)

**Keywords:** hepatocellular carcinoma, skeletal muscle depletion, sarcopenia, prognostic factor, sorafenib

## Abstract

The aim of this study was to determine whether skeletal muscle depletion predicts the prognosis of patients with hepatocellular carcinoma (HCC) that is being treated with sorafenib. We evaluated 40 consecutive HCC patients who received sorafenib treatment. The skeletal muscle cross-sectional area was measured by computed tomography at the third lumbar vertebra (L3), from which the L3 skeletal muscle index (L3 SMI) was obtained. The factors contributing to overall survival, sorafenib dose reduction, and discontinuation of sorafenib were analyzed using the Cox proportional hazards model. L3 SMI (*p* = 0.020) and log (α-fetoprotein (AFP)) (*p* = 0.010) were identified as independent prognostic factors in HCC patients treated with sorafenib. The initial dose of sorafenib (*p* = 0.008) was an independent risk factor for sorafenib dose reduction, and log (AFP) (*p* = 0.008) was the only significant risk factor for the discontinuation of this drug. L3 SMI was not a risk factor for either dose reduction (*p* = 0.423) or the discontinuation (*p* = 0.132) of sorafenib. A multiple linear regression analysis determined the following relationship between skeletal muscle mass (assessed as L3 SMI) and the explanatory factors: L3 SMI = −0.1896 × (Age) − 10.3441 × (Child-Pugh score) − 9.3922 × (log (AFP)) + 1.6139 × (log (AFP)) × (Child-Pugh score) + 112.9166. Skeletal muscle depletion is inversely associated with age, Child-Pugh score, and log (AFP). Moreover, it is an independent prognostic factor for HCC patients treated with sorafenib.

## 1. Introduction

Hepatocellular carcinoma (HCC), which usually develops in the cirrhotic liver, is one of the most common malignancies worldwide [1,2]. It is essential to be able to predict the patient’s prognosis in order to select the most adequate treatments for malignancies such as HCC. However, outcome prediction is extremely complicated for HCC because the liver functional reserve influences prognosis, sometimes to a greater extent than does the progression of HCC itself [3]. Further, the estimation of tumor stage alone is insufficient for predicting the prognosis of HCC since this malignancy very frequently recurs after curative treatment [4]. In order to predict the prognosis of HCC more precisely, several prognostic staging systems have been developed, such as the Barcelona Clinic Liver Cancer (BCLC) [5], Cancer of the Liver Italian Program (CLIP) [6], and Japan Integrated Staging (JIS) [7] systems. These staging systems are suitable for predicting HCC prognosis because they incorporate both tumor stages and liver functional reserves. However, it is also vital to continually make efforts to identify additional useful and simple factors that predict the prognosis of HCC.

Skeletal muscle depletion, or sarcopenia, was initially defined as the loss of skeletal muscle mass that occurs with aging [8]. Skeletal muscle depletion has been critically implicated in a variety of pathological conditions, such as malnutrition, advanced organ failure, and inflammatory disease [9]. It has also been identified as a poor prognostic factor for various types of human malignancies [10,11,12,13]. Particularly, cases of liver cirrhosis and HCC are frequently complicated by sarcopenia, which is associated with a poor prognosis for these diseases [14,15,16,17]. In a study of 217 consecutive patients with all stages of HCC, we found a strong relationship between skeletal muscle depletion and poor prognosis [16]. These previous findings, including our own data, suggest that skeletal muscle mass could be a useful and objective indicator for predicting the prognosis of patients with HCC.

Sorafenib (Nexavar, Bayer HealthCare Pharmaceuticals, Leverkusen, Germany) is the first orally active multi-kinase inhibitor that has been confirmed to be efficacious against advanced HCC [18,19]. Sorafenib has become the standard first-line treatment for patients with advanced HCC, but it sometimes causes severe side effects, such as hand-foot syndrome and liver dysfunction, which may limit the patient’s ability to continue full-dose treatment [20,21]. A recent clinical trial showed that sarcopenia predicts early dose-limiting toxicities in patients who have advanced HCC with Child-Pugh A liver cirrhosis [20]. Therefore, we hypothesized that the poor prognosis of patients with sarcopenia and HCC might be caused by early dose reductions or the discontinuation of sorafenib due to its increased toxicity.

In the present study, we used computed tomography (CT) to measure the skeletal muscle cross-sectional areas of patients with HCC just before they began to receive treatment with sorafenib. Further, we designed a retrospective analysis to identify factors, including skeletal muscle mass, that could influence the overall survival of consecutive HCC patients treated with sorafenib. Finally, we have discussed the mechanisms by which skeletal muscle depletion affects mortality in HCC and the actions that should be taken to prevent sarcopenia in HCC patients treated with sorafenib

## 2. Results

### 2.1. Baseline Characteristics and Laboratory Data

The baseline characteristics and laboratory data of the 40 patients (34 men and 6 women; median age, 67 years) are shown in Table 1. The median L3 skeletal muscle index (SMI) was 41.9 cm^2^/m^2^ (range, 24.9–64.5 cm^2^/m^2^), and the median duration of follow-up was 12.2 months (range, 0.4–33.7 months). Three patients had already started to receive sorafenib when the disease had been in an earlier stage (Stage 0). Thirty-nine patients had received other treatments before they began to take sorafenib. Twenty-nine patients received combination treatments with sorafenib, 19 of whom received transcatheter arterial chemoembolization (TACE), 4 of whom received radiation therapy, and 6 of whom received both of these treatments.

**Table 1:** Baseline demographic and clinical characteristics.

Variables	*n =* 40
Sex (male/female)	34/6
Age (years)	67 (27–86)
Etiology (HBV/HCV/other)	13/20/7
Follow-up period (months)	12.2 (0.4–33.7)
BMI (kg/m^2^)	21.7 (16.5–30.1)
L3 SMI (cm^2^/m^2^)	41.9 (24.9–64.5)
Child-Pugh score (5/6)	27/13
Albumin (g/dL)	3.6 (2.8–4.6)
ALT (IU/L)	26.5 (8–65)
T-Bil (mg/dL)	0.8 (0.4–3.5)
PLT (×10^4^/μL)	11.6 (2.1–61.3)
PT (%)	89.5 (66–120)
AFP (ng/dL)	100.2 (2.3–211, 459.8)
PIVKA-II (mAU/mL)	253 (15–254, 450)
Stage (0/I/II/III/IVA/IVB)	3/0/4/9/4/20
Prior treatment (yes/no)	39/1
Combination treatment (TACE/RT/TACE + RT/no)	19/4/6/11
Initial dose of sorafenib per day (mg) (100/200/400/800)	1/13/24/2

Values are presented as median (range). HBV, hepatitis B virus; HCV, hepatitis C virus; BMI, body mass index; L3 SMI, third lumbar vertebra skeletal muscle index; ALT, alanine aminotransferase; T-Bil, total bilirubin; PLT, platelet count; PT, Prothrombin time; AFP, α-fetoprotein; PIVKA-II, protein induced by vitamin K absence or antagonists-II; TACE, transarterial chemoembolization; RT, radiation therapy.

### 2.2. Effects of Aging on Skeletal Muscle Depletion

In order to evaluate the influence of aging on skeletal muscle depletion, we performed a paired *t-*test of the 36 cases in which CT was performed more than twice during the trial. This analysis showed that L3 SMI declined by an average of 2.7 cm^2^/m^2^ at an interval of 155 days (*p* < 0.0001).

### 2.3. Possible Prognostic Factors for HCC Patients

**Figure 1:**
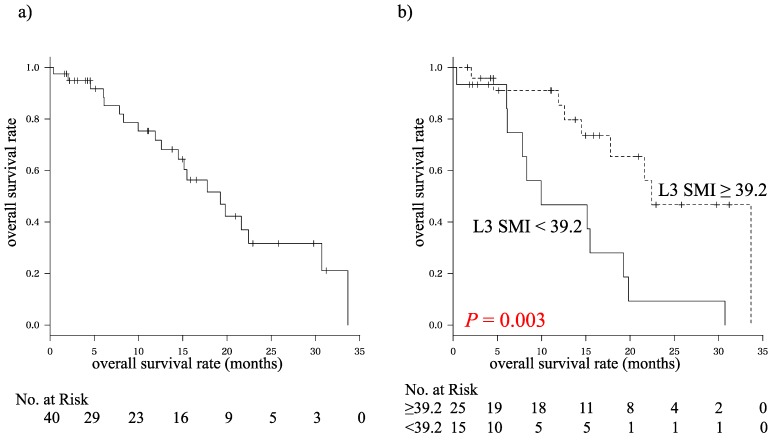
Kaplan–Meier curves for overall survival time in (**a**) all patients and (**b**) subgroups based on L3 skeletal muscle index (L3 SMI) (<39.2 and ≥39.2 (cm^2^/m^2^)).

Of the 40 patients, 20 died during the study period. Overall survival rates at one, two, and three years were 71.7%, 31.7%, and 21.2%, respectively, and the median duration of overall survival was 19.5 months (Figure 1a). In regards to response rates, 0, 3, 9 and 28 of the enrolled patients were classified as having complete response (0%), partial response (8.0%), stable disease (22.0%), and progressive disease (70.0%), respectively.

We analyzed possible prognostic factors for HCC using the Cox proportional hazards model. Among the 10 variables listed in Table 2, L3 SMI (*p* = 0.020) and log (α-fetoprotein (AFP)) (*p* = 0.008) were significantly associated with the overall survival of patients with HCC in univariate analyses. Both L3 SMI (*p* = 0.020) and log (AFP) (*p* = 0.010) were also independent prognostic factors for HCC in our multivariate analysis. Furthermore, Kaplan–Meier analysis showed that overall survival was significantly shorter in patients with lower L3 SMI (≤39.2 cm^2^/m^2^, *p* = 0.003, Figure 1b). The 39.2 cm^2^/m^2^ cut-off was selected to maximize the difference between the two survival curves, using a statistical analysis reported by Prado *et al.* [10]. In the present study, 15 patients had a L3 SMI of less than 39.2 cm^2^/m^2^ and 25 patients had an L3 SMI of more than 39.2 cm^2^/m^2^.

**Table 2:** Univariate and multivariate analyses of possible risk factors for overall survival of hepatocellular carcinoma patients by the Cox proportional hazards model.

Variable	Univariate Analysis		Multivariate Analysis	
	HR (95% CI)	*p* Value	HR (95% CI)	*p* Value
Age (years)	0.976 (0.944–1.008)	0.140		
BMI (kg/m^2^)	0.863 (0.715–1.042)	0.127		
L3 SMI (cm^2^/m^2^)	0.904 (0.830–0.984)	0.020	0.909 (0.836–0.985)	0.020
Child-Pugh score	0.840 (0.312–2.265)	0.730		
Albumin (g/dL)	0.556 (0.180–1.722)	0.309		
PLT (×10^4^/mL)	1.049 (0.992–1.111)	0.096		
log (AFP)	1.793 (1.162–2.767)	0.008	1.274 (1.0580–1.534)	0.010
log (PIVKA-II)	1.024 (0.857–1.224)	0.794		
Stage (IVB *vs.* others)	1.062 (0.407–2.770)	0.902		
Combination therapy (yes *vs.* no)	0.533 (0.188–1.509)	0.236		

BMI, body mass index; L3 SMI, third lumbar vertebra skeletal muscle index; PLT, platelet count; AFP, α-fetoprotein; PIVKA-II, protein induced by vitamin K absence or antagonists-II.

### 2.4. Possible Risk Factors for Sorafenib Dose Reduction or Discontinuation Due to Toxicity

During this study, the dose of sorafenib was reduced for 32 patients. Doses were reduced because of hand-foot syndrome in 6 patients, liver dysfunction in 5 patients, the progression of liver cirrhosis in 4 patients, deteriorating general condition in 4 patients, appetite loss in 3 patients, diarrhea in 2 patients, and assorted other reasons in the remaining 8 patients. As shown in Table 3, only the initial dose of sorafenib (*p* = 0.008) was significantly associated with sorafenib dose reduction.

**Table 3:** Univariate and multivariate analyses of possible risk factors for dose reduction of sorafenib due to its toxicity by Cox proportional hazards model.

Variable	Univariate Analysis	
	HR (95% CI)	*p* Value
Age (years)	0.980 (0.954–1.007)	0.143
BMI (kg/m^2^)	0.983 (0.864–1.119)	0.797
L3 SMI	0.979 (0.928–1.032)	0.423
Child-Pugh score	0.844 (0.394–1.807)	0.662
Albumin (g/dL)	0.789 (0.338–1.840)	0.583
PLT (×10^4^/mL)	1.011 (0.975–1.049)	0.554
PT (%)	0.995 (0.971–1.019)	0.673
T-Bil (mg/dL)	1.696 (0.782–3.681)	0.181
log (AFP)	1.311 (0.980–1.753)	0.069
log (PIVKA-II)	1.010 (0.891–1.145)	0.876
Stage (IVB *vs.* others)	1.560 (0.738–3.295)	0.244
Initial dose of sorafenib (mg)	1.005 (1.001–1.009)	0.008

BMI, body mass index; L3 SMI, third lumbar vertebra skeletal muscle index; PLT, platelet count; PT, Prothrombin time; T-Bil, total bilirubin; AFP, α-fetoprotein; PIVKA-II, protein induced by vitamin K absence or antagonists-II.

With respect to drug withdrawal, 28 patients were forced to discontinue sorafenib during this study. Ten patients discontinued sorafenib because of the deterioration of their general condition, 7 because of the progression of liver cirrhosis, 4 because of hand-foot syndrome, 3 because of disease progression, and the remaining 4 because of other factors. Among the variables listed in Table 4, log (AFP) (*p* = 0.008) was the only significant risk factor for the discontinuation of sorafenib. Neither sorafenib dose reduction (*p* = 0.423) nor the discontinuation of sorafenib (*p* = 0.132) was significantly associated with the value of L3 SMI (Table 3 and Table 4).

**Table 4:** Univariate and multivariate analyses of possible risk factors for discontinuation of sorafenib due to its toxicity by Cox proportional hazards model.

Variable	Univariate Analysis	
	HR (95% CI)	*p* Value
Age (years)	0.975 (0.945–1.006)	0.108
BMI (kg/m^2^)	0.927 (0.802–1.072)	0.307
L3 SMI	0.951 (0.891–1.015)	0.132
Child-Pugh score	0.987 (0.448–2.176)	0.974
Albumin (g/dL)	0.743 (0.297–1.860)	0.526
PLT (×10^4^/mL)	1.017 (0.975–1.062)	0.436
PT (%)	0.998 (0.973–1.023)	0.859
T-Bil (mg/dL)	1.530 (0.803–2.914)	0.196
log (AFP)	1.222 (1.055–1.415)	0.008
log (PIVKA-II)	1.044 (0.904–1.204)	0.560
Stage (IVB *vs.* others)	1.559 (0.727–3.346)	0.254
Initial dose of sorafenib (mg)	1.004 (0.999–1.008)	0.077

BMI, body mass index; L3 SMI, third lumbar vertebra skeletal muscle index; PLT, platelet count; PT, Prothrombin time; T-Bil, total bilirubin; AFP, α-fetoprotein; PIVKA-II, protein induced by vitamin K absence or antagonists-II.

### 2.5. Multiple Linear Regression Analysis of L3 SMI and Significant Explanatory Valuables

Loss of skeletal muscle mass is primarily a consequence of aging and secondarily a consequence of pathological conditions, such as malnutrition and complications of malignancy [9]. Men and women have different absolute skeletal muscle masses and rates of skeletal muscle mass reduction with increasing age [22,23]. Prompted by these earlier findings, we conducted a multiple regression analysis to examine the relationships between L3 SMI and 4 explanatory variables: age, Child-Pugh score, log (AFP), and sex. At first, we included each explanatory variable and all two-way interactions between explanatory variables within the multiple regressions. Subsequently, we removed the term with the highest *p*-value one by one until all remaining terms were significant (*p* < 0.05). In other words, we employed a backwards variable selection procedure. As detailed in Table 5, three individual explanatory variables and the interaction between log (AFP) and Child-Pugh score remained significant. The final result of the multiple regression analysis was as follows (with an intercept of 112.9166):
L3 SMI =−0.1896× (Age)
−10.3441(Child-Pugh score)
−9.3922× (log (AFP))
+1.6139× (log (AFP)) × (Child-Pugh score)
+112.9166



**Table 5:** Multiple linear regression analysis with L3 skeletal muscle index (SMI) and significantly explanatory valuables including each other’s interactions.

Variables	Std. Coefficient	Std. Error	*t* Value	*p* Value
Intercept	112.9166	20.9930	5.38	<0.0001
Age	−0.1896	0.0758	−2.50	0.017
Child-Pugh score	−10.3441	3.9395	−2.63	0.013
log (AFP)	−9.3922	3.6142	−2.60	0.014
log (AFP): Child-Pugh score	1.6139	0.6657	2.42	0.021

Multiple R-squared: 0.338, F-statistic: 4.46 on 4 and 35 degree of freedom, *p* value = 0.00512.

This regression formula indicated that the level of L3 SMI was inversely associated with aging, worsening liver functional reserve, and increasing AFP levels.

## 3. Discussion

The results of the present study provide the first clear evidence that L3 SMI, an indicator of skeletal muscle mass or sarcopenia, is an independent prognostic factor for patients who have HCC and are being treated with sorafenib. These findings agree with previous studies that have shown significant relationships between skeletal muscle depletion and poor prognosis in patients with various clinical stages of HCC [14,15,16,17].

In the present study, we also obtained a regression equation that can be used to estimate L3 SMI from three explanatory factors: age, Child-Pugh score, and log (AFP). It has previously been reported that these factors may be associated with loss of skeletal muscle mass [9,22,23]. This analysis indicates that L3 SMI decreases in correspondence with increasing age, worsening liver functional reserve, and elevated AFP levels in patients with HCC who receive sorafenib. The interaction effect between log (AFP) and Child-Pugh score was quite small and can be ignored because of the large differences between the coefficient of the interaction effect and the coefficients of the associated main effects. Indeed, the combined value of the log (AFP) term, the Child-Pugh score term, and the interaction term (1.6139 × (log (AFP)) × (Child-Pugh score) − 10.3441 × (Child-Pugh score) − 9.3922 × (log (AFP))) was far less than 0 in all patients (ranging from −66.46 to −52.82). The regression equation could present the key to explaining the association between skeletal muscle depletion and poor prognosis because liver functional reserve and AFP levels are both well-known prognostic factors for HCC [3]. Moreover, L3 SMI was affected by ageing, which itself affects the prognosis of patients with most medical conditions, even though existing prognostic staging systems for HCC (such as BCLC, CLIP, and JIS) do not include an aging parameter [5,6,7]. Thus, the evaluation of skeletal muscle mass using CT imaging could present a useful alternative method of predicting the prognosis of patients with HCC.

A recent clinical study reported that sarcopenia predicts early dose-limiting sorafenib toxicities, which might be involved in dose reductions or the discontinuation of this drug [20]. Therefore, we initially speculated that the more favorable prognosis of HCC patients without skeletal muscle depletion was associated with the greater extent of continuous sorafenib treatment that they received. However, contrary to this expectation, the loss of L3 SMI did not affect dose reductions or the discontinuation of sorafenib in the present study. We consider this finding to be explained by the initial dose of sorafenib, which was comparatively small. Specifically, the dose was less than 400 mg per day in most cases (95%). These low doses might strengthen the patients’ adherence to sorafenib and, consequently, improve the prognosis of these patients in the present study. On the other hand, it should be noted that the initial dose was a risk factor for dose reduction. Further, we found that log (AFP) was a risk factor for the discontinuation of sorafenib. Therefore, it is necessary to be especially careful when facing cases of sorafenib-treated HCC that involve initial doses of 800 mg per day or high AFP levels. In such cases, adverse events that require dose reductions or discontinuation may occur with a high probability.

Importantly, the survival outcomes of the present study were quite favorable as compared with a previous phase III trial by Llovet *et al.* In the present and previous studies, the median overall survival times were 19.5 and 10.7 months, respectively, and the survival rates at 1 year were 71.7% and 44.0%, respectively [18]. The positive outcomes in our study may result from several factors. First, sorafenib was introduced at earlier tumor stage in some cases. Indeed, in three cases, it was used before obvious evidence of recurrent lesion had emerged, although pathological assessments of the resected specimen revealed either vascular invasion or focal positivity. Second, a combination treatment using sorafenib was administered in 29 cases (72.5%). It is also interesting that, among the patients who received combination treatment, six cases (20.6%) showed increased levels of skeletal muscle mass during this study, whereas only one case (9.1%) showed this phenomenon in the group of patients who were treated with sorafenib alone. Combined treatment with sorafenib and TACE has a strong scientific rationale because the ischemia that is induced by TACE results in local and systemic increases in vascular endothelial growth factor (VEGF), whereas sorafenib inhibits the activity of the VEGF receptor [24]. Several meta-analyses have shown a positive outcome in HCC patients treated with TACE and sorafenib, as compared with HCC patients treated with TACE alone [25,26,27]. Currently, several randomized, controlled phase III trials of the safety and efficacy of sorafenib-based combination treatment are ongoing. These basic and clinical studies may provide new evidence that combination treatment with sorafenib is a promising approach for improving the prognosis of patients with HCC. However, additional careful investigation is necessary to verify the safety and efficacy of this combination treatment.

As demonstrated in the present study, skeletal muscle depletion seems to be an inevitable consequence of aging. On the other hand, it is possible that the prevention of skeletal muscle depletion might be an effective strategy for improving the prognosis of HCC; this approach should be considered further. Two possible interventions could prevent sarcopenia: nutritional and exercise therapies. Both of these interventions have been shown to improve outcomes for cirrhotic patients [28,29]. In particular, oral supplementation with branched-chain amino acids (BCAA; leucine, isoleucine and valine) is an effective nutritional therapy that can ameliorate protein energy malnutrition and improve event-free survival in cirrhotic patients [30,31,32]. BCAA supplementation is one of the most promising methods because it has been observed to improve the survival of sarcopenic patients with liver cirrhosis [33]. In a study of patients with alcoholic cirrhosis, Tsien C *et al.* demonstrated that impaired mTOR1 signaling and increased autophagy in skeletal muscle, which contribute progression of sarcopenia, are reversed by BCAA enriched with leucine [34]. Furthermore, the combination of a low intensity exercise and leucine-rich essential amino acid protein administration is effective for enhancing not only muscle strength, but also muscle mass and walking speed in sarcopenic women [35]. These reports suggest that BCAA supplementation, taking proper exercise, and the combination of these interventions might be effective for preventing sarcopenia. Future large-scale interventional studies should be conducted to determine whether nutritional and exercise therapies can prevent skeletal muscle depletion and possibly improve the prognosis of patients with HCC.

In summary, this study provides the first demonstration that L3 SMI, an indicator of skeletal muscle mass, is independently associated with poor prognosis among patients who have HCC and receive sorafenib treatment. In contrast, we found that L3 SMI was not a significant risk factor for dose reduction or the discontinuation of sorafenib. In addition, a multiple linear regression analysis suggested that L3 SMI was inversely associated with age, log (AFP), and Child-Pugh score, which indicated that these L3 SMI-correlated clinical features are also critical prognostic factors for patients with HCC. In conclusion, the evaluation of skeletal muscle mass using CT imaging could present a useful method of predicting the prognosis of patients with HCC, including those who are treated with sorafenib.

## 4. Experimental Section

### 4.1. Patients, Treatment, and Follow-Up Strategy

We evaluated 40 consecutive patients with HCC that was treated with sorafenib at our hospital between May 2009 and February 2014. Tumor stage was defined according to the staging system of the Liver Cancer Study Group of Japan [36]. HCC nodules were detected using imaging modalities including dynamic CT, dynamic magnetic resonance imaging (MRI), and abdominal arteriography. HCC was diagnosed on the basis of a typical hypervascular tumor stain on angiography and typical dynamic study findings of enhanced staining in the early phase and attenuation in the delayed phase. The objective of sorafenib introduction was determined according to the Clinical Practice Guidelines for HCC issued by the Japan Society of Hepatology [37]. Each patient’s response was judged according to the Response Evaluation Criteria in Cancer of the Liver [38], which is an appropriate system for assessment of post-therapeutic response to sorafenib for HCC [39]. Overall survival was defined as the interval from the date of sorafenib introduction to the date of death, or until July 2014 for surviving patients. All study participants provided verbal informed consent, which was considered sufficient because this study followed an observational research design that did not require new human biological specimens, and instead relied only on preexisting materials. This study protocol was determined by following the Ethical Guidelines for Epidemiological Research (Ministry of Education, Culture, Sports, Science and Technology and Ministry of Health, Labour and Welfare in Japan, http://www.niph.go.jp/wadai/ekigakurinri/guidelines.pdf). The study design—including this consent procedure—was approved by the ethics committee of the Gifu University School of Medicine in 1 April 2014.

### 4.2. Image Analysis of Skeletal Muscle Mass

Skeletal muscle area was measured using a CT image that had been taken solely for the purpose of diagnosing HCC just before sorafenib was initiated. A transverse CT image at the third lumbar vertebra (L3) in the inferior direction was assessed as has been described previously [10]. The muscles in the L3 region (containing the psoas, erector spinae, quadratus lumborum, transversus abdominis, external and internal obliques, and rectus abdominis) were analyzed using SliceOmatic software version 5.0 (Tomovision, Montreal, QC, Canada), which enables specific tissue demarcation using Hounsfield unit (HU) thresholds. The muscles were quantified within a HU range of −29 to +150 HU [40], and tissue boundaries were manually corrected as needed. The cross-sectional areas of muscle (cm^2^) at the L3 level were computed from each image and normalized by the square of the height (m^2^) to obtain the L3 skeletal muscle index (L3 SMI, cm^2^/m^2^), which was used as an indicator of skeletal muscle mass [16]. The progression of skeletal muscle depletion was evaluated by comparing the values of L3 SMI for cases in which CT was performed more than twice during the trial.

### 4.3. Statistical Analysis

Overall survival was estimated using the Kaplan–Meier method, and differences between curves were evaluated using the log-rank test. Prognostic factors affecting overall survival were estimated using the Cox proportional hazards model. Parameters determined to be significant according to univariate analyses were then included in a multivariate analysis. Parameters affecting the interval from the date of sorafenib introduction to the date of dose reduction or discontinuation due to sorafenib toxicity were also estimated in the same manner. Statistical significance was defined as *p* < 0.05. All statistical analyses were carried out using R version 3.1.1 (R Foundation for Statistical Computing, Vienna, Austria. URL: http://www.R-project.org/).
